# Effects of Gouge Fill on Elastic Wave Propagation in Equivalent Continuum Jointed Rock Mass

**DOI:** 10.3390/ma14123173

**Published:** 2021-06-09

**Authors:** Ji-Won Kim, Song-Hun Chong, Gye-Chun Cho

**Affiliations:** 1 Radioactive Waste Disposal Research Division, Korea Atomic Energy Research Institute (KAERI), 111 Daedeok-daero 989 Beon-gil, Yuseong-gu, Daejeon 34057, Korea; jwk@kaeri.re.kr; 2 Department of Civil Engineering, Sunchon National University, 225 Jungang-ro, Suncheon 57922, Jeollanam-do, Korea; shchong@scnu.ac.kr; 3 Department of Civil and Environmental Engineering, Korea Advanced Institute of Science and Technology, 291 Daehak-ro, Yuseong-gu, Daejeon 34141, Korea

**Keywords:** elastic wave velocity, gouge fill, jointed rock, quasi-static resonant column test

## Abstract

The presence of gouge in rock joints significantly affects the physical and mechanical properties of the host rock mass. Wave-based exploration techniques have been widely used to investigate the effects of gouge fill on rock mass properties. Previous research on wave propagation in gouge-filled joints focused on analytical and theoretical methods. The lack of experimental methods for multiple rock joint systems, however, has limited the verification potential of the proposed models. In this study, the effects of gouge material and thickness on wave propagation in equivalent continuum jointed rocks are investigated using a quasi-static resonant column test. Gouge-filled rock specimens are simulated using stacked granite rock discs. Sand and clay gouge fills of 2 and 5 mm thicknesses are tested to investigate the effects of gouge material and thickness. Comprehensive analyses of the effects of gouge thickness are conducted using homogeneous isotropic acetal gouge fills of known thickness. The results show that gouge fill leads to changes in wave velocity, which depend on the characteristics of the gouge fill. The results also show that particulate soil gouge is susceptible to preloading effects that cause permanent changes in the soil fabric and contact geometry and that increased gouge thickness causes a more significant stiffness contribution of the gouge material properties to the overall stiffness of the equivalent continuum specimen. The normal and shear joint stiffnesses for different gouge fill conditions are calculated from the experimental results using the equivalent continuum model and suggested as input parameters for numerical analysis.

## 1. Introduction

The presence and nature of rock joints govern the physical behavior of rock masses and affect elastic wave propagation and attenuation [1,2,3,4]. Natural rock joints are filled with gouge material ranging from fluids (e.g., water or oil) to viscoelastic particulate soils (e.g., sand, silt, and clay). The effects of gouge fill on wave propagation in jointed rock masses have been examined extensively in analytical and experimental studies [5,6,7,8,9,10,11,12]. Prior research noted that the presence of gouge fill greatly influences the mechanical behavior and seismic response of the jointed rock mass for single and multiple joints. Zhu et al. (2011) [13] suggested analytical solutions for wave propagation in single and multiple rock joints with viscoelastic gouge fills and conducted modified split Hopkinson pressure bar experiments on single sand-filled joints. Li et al. (2014) [14] proposed a thin-layer interface model by modeling gouge fills as thick-layer interfaces between adjacent rocks to analyze oblique wave propagation in jointed rock masses. However, previous experimental studies on gouge fill joints focused on single joints, and the lack of experimental methods for testing jointed rocks with multiple joints and joint sets limits the verification potential of the proposed models.

Rock joints typically exist as joint sets with multiple parallel joints, and the spatial scale of the wave propagation is much greater than that of the joint spacing. In this context, the equivalent medium model has been used to calculate the effective modulus of jointed rock masses by incorporating the effect of rock joints on rock mass properties [15,16,17]. The long-wavelength equivalent continuum model can oversimplify the discrete joint characteristics of jointed rock mass and is inapplicable to sparsely spaced joints. However, the model is valid for typical field scenarios where the wavelength of the propagating wave is much longer than the spatial heterogeneity of the rock. Based on this equivalent medium concept, Fratta and Santamarina (2002) [18] developed the quasi-static resonant column (QSRC) test, which can measure long-wavelength shear wave propagation in jointed rock masses. The QSRC test uses stacked rock discs to simulate rock masses with regularly spaced joints at the laboratory scale. The jointed rock mass specimen is in an equivalent continuum, where the wavelength of the propagating wave is much greater than the length scale of the joint spacing. Previous studies using the QSRC test were conducted under different gouge conditions. Fratta and Santamarina (2002) examined the effects of gouge thickness on the shear wave velocity and damping ratio using kaolinite fills with thicknesses ranging from 0 to 2.5 mm. Cha et al. (2009) [19] added a longitudinal wave measurement system to the QSRC test and tested the effects of gouge fill using sand and clay fills having thicknesses ranging from 0 to 1.5 mm. Chong et al. (2014) [20] developed the rock mass dynamic test (RMDT), which can test the strain-dependent wave propagation in QSRC test specimens, and Kim et al. (2018) [21] used the QSRC test and RMDT setups to test the effects of grouted rock joints. Chong et al. (2020) [22] simulated the QSRC setup using discrete element numerical analysis and investigated the long-wavelength propagation in jointed rock mass for different materials and stress conditions. However, the examination of larger gouge thicknesses and suggestions for modeling gouge fills in numerical analysis require more in-depth studies.

The effects of rock joint infill and thickness on wave propagation in equivalent continuum jointed rocks are investigated based on the QSRC test. Experimental studies using sand and clay gouge fills with 2 and 5 mm fill thicknesses are performed to investigate the effects of cohesive and frictional soil gouge fills and fill thickness. The effects of gouge thickness are further analyzed using homogeneous isotropic acetal discs of known thickness. The normal and shear joint stiffnesses for different gouge fill conditions are calculated from the experimental results using the equivalent continuum model and suggested as input parameters for numerical analysis.

## 2. Experimental Method

**Figure 1:**
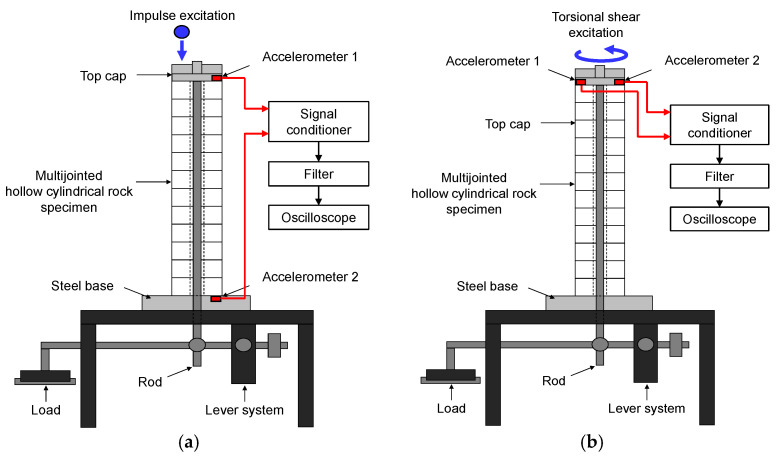
QSRC test setup: (**a**) longitudinal wave setup; (**b**) shear wave setup. Excitation method and directions are shown in red.

The QSRC test setup was used to measure long-wavelength longitudinal and shear elastic wave propagations in jointed rock specimens. The QSRC test uses quasi-static elastic deformations to generate longitudinal and torsional shear excitations in a jointed rock mass specimen to measure the long-wavelength longitudinal and shear velocities, as shown in Figure 1. Twelve hollow cylindrical rock discs were stacked to simulate jointed rock columns in equivalent continuum. Previous studies noted that nine or more discs are required to avoid Brillouin dispersion and to achieve long-wavelength propagation in a stacked rock column [19]. The rock specimens were placed on a high-impedance steel base to simulate fixed-free boundary conditions. An aluminum top cap and a loading rod were placed on top of the specimen for vertical loading. The loading rod was connected to a lever system that multiplies the load applied on the specimen by a factor of 10. Two accelerometers were used to measure the longitudinal and shear wave velocities. For longitudinal wave measurements, the accelerometers were installed at the top and bottom of the specimen. Longitudinal excitation was simulated by dropping a steel ball bearing onto the top of the specimen. The longitudinal wave velocity was calculated using the point-source travel-time method:

(1) $$V_{P}{}^{QSRC}=\frac{L}{\Delta t},$$

where *L* is the height of the specimen and Δ*t* is the travel time measured between the two accelerometers.

For shear wave measurements, two accelerometers were installed at diametrically opposite locations on the top cap. Torsional shear excitation was simulated by breaking a brittle 0.5 mm pencil lead, which released the specimen from a static state. The time-domain signals from the two accelerometers were added to remove the flexural responses in the slender rock specimen. The added signals were transformed into the frequency domain using a fast Fourier transformation. The shear wave velocity was calculated by multiplying the resonant frequency by four times the specimen length, considering the fixed-free boundary condition, using the following equation:

(2) $$V_{S}{}^{QSRC}=4Lf_{n},$$

where *L* is the height and *f_n_* is the resonant frequency of the specimen, respectively.

**Figure 2:**
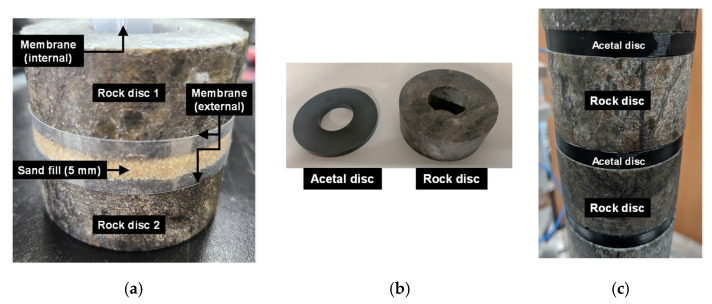
Preparation of jointed rock specimens with gouge fill: (**a**) 5 mm Jumunjin sand fill specimen with internal and external plastic membranes; (**b**) rock and 5 mm acetal discs; (**c**) 5 mm acetal fill specimen with alternating acetal and rock discs.

First, the effects of the gouge fill material and fill thickness were examined using particulate soil gouge fills. Internal and external plastic membranes were installed between each disc to hold the gouge fill material, as shown in Figure 2a. Dry Jumunjin sand and kaolinite gouge fills having thicknesses of 2 and 5 mm were used to represent the frictional and cohesive materials, respectively. The mass of each rock disc and membrane was measured before and after the addition of the gouge fill material to ensure the homogeneity of the joints. The average mass of sand fill per joint was 4.95 g for 2 mm and 12.36 g for 5 mm joint fill thickness, respectively. The average mass of clay fill per joint was 1.44 g for 2 mm and 3.45 g for 5 mm joint fill thicknesses, respectively. Second, the effects of gouge thickness were further analyzed using homogeneous isotropic acetal discs of known thickness. Laser-cut 1, 2, 3, and 5 mm acetal discs were used to further examine the effects of absolute gouge fill thickness, as shown in Figure 2b. Longitudinal and shear wave velocity measurements were taken for loading and unloading steps from 50 to 250 kPa and 250 to 50 kPa axial stresses at 50 kPa intervals. The properties of the rock specimen and gouge materials used in this study are listed in Table 1, and the experimental cases are listed in Table 2. The intact P-wave and rod wave velocities of the intact rock specimen were obtained from free–free resonant column tests and point-source travel-time method. The intact S-wave velocities of the intact rock specimen were calculated from the theoretical relationship [23]:

(3) $$\upsilon =\frac{V_{P}^{2}-2V_{S}^{2}}{2(V_{P}^{2}-V_{S}^{2})}$$

where *V_S_* is the shear wave velocity, *V_P_* is the longitudinal wave velocity, and *υ* is Poisson’s ratio. The Poisson’s ratio was indirectly calculated from the rod wave velocity and P-wave velocity using the following equation:

(4) $$\frac{V_{rod}^{2}}{V_{P}^{2}}=\frac{\left(1+\nu \right)\left(1-2\nu \right)}{\left(1-\nu \right)}$$

## 3. Experimental Results

### 3.1. Effects of Soil Gouge Fill

**Figure 3:**
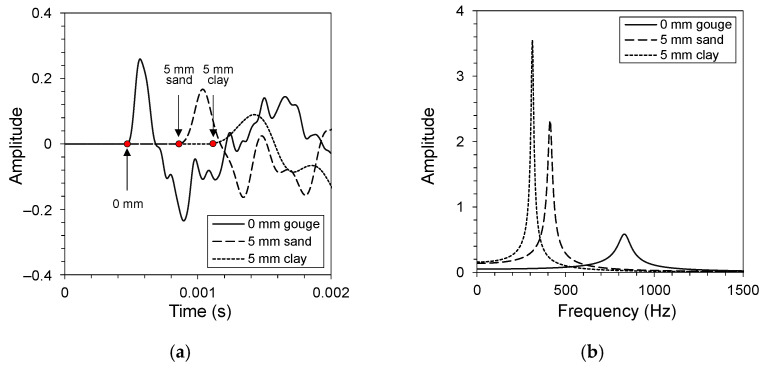
Data analysis of long-wavelength velocity for jointed rock specimens with different gouge fill conditions subjected to 250 kPa normal stress. (**a**) Time-domain data obtained from longitudinal QSRC tests. The arrival time of each time-domain signal is highlighted using red dots and arrows. (**b**) Frequency-domain data obtained from torsional shear QSRC tests.

Figure 3 presents the changes in the time- and frequency-domain wave data from the QSRC tests on clean-cut jointed rock and soil gouge fill rock specimens. The time-domain data measured at the bottom of the specimen were normalized by the maximum amplitude of their respective impact sources measured on top of the specimen. The time-domain data was shifted such that the time of impact of each test was 0 s. This allows direct comparisons between the received time-domain data. The longitudinal QSRC test results displayed decreased amplitude and longer travel time with increased gouge thickness and softer gouge fill, which implies that less energy is transmitted through the jointed rock mass with gouge fill. The presence of gouge fill resulted in decreased contact joint stiffness and served as additional sources of attenuation. The frequency domain data displayed a decreased resonant frequency and increased amplitude with increased gouge thickness and softer gouge fill. Torsional shear waves propagated perpendicular to the direction of loading, and softer gouge fill resulted in reduced joint stiffness and improved wave transmission.

**Figure 4:**
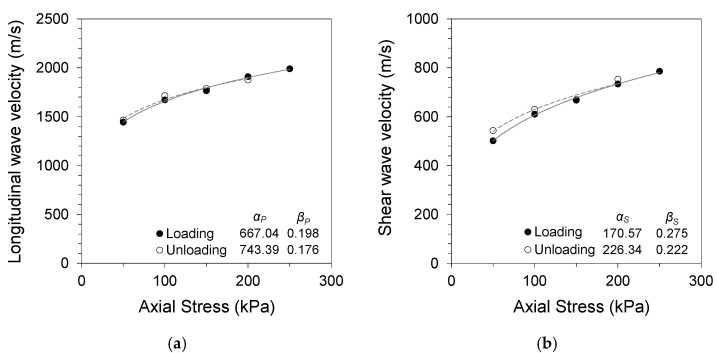
Specimen without gouge (0 mm): (**a**) longitudinal wave velocity against normal stress; (**b**) shear wave velocity against normal stress.

**Figure 5:**
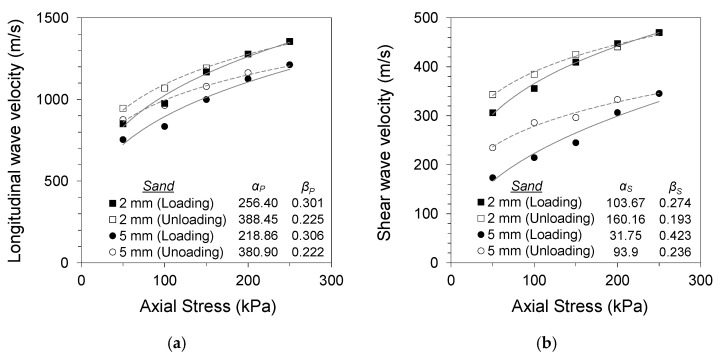
Specimen with Jumunjin sand gouge (2, 5 mm): (**a**) longitudinal wave velocity against normal stress; (**b**) shear wave velocity against normal stress.

**Figure 6:**
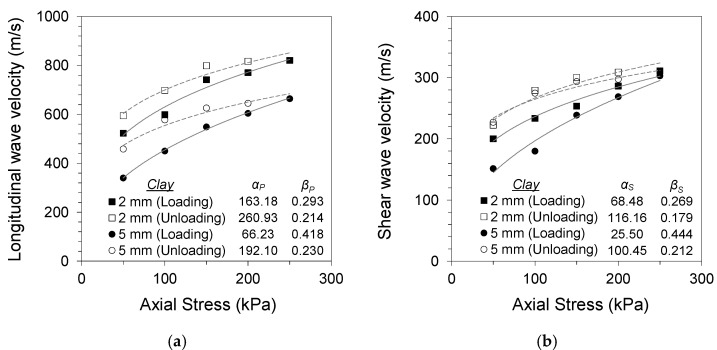
Specimen with kaolinite clay gouge (2, 5 mm): (**a**) longitudinal wave velocity against normal stress; (**b**) shear wave velocity against normal stress.

The small strain stiffness in jointed rocks can be expressed using the Hertzian power function in terms of the applied axial stress using the following equations:

(5) $$V_{P}=\alpha_{P}{\left(\frac{\sigma_{n}}{1kPa}\right)}^{\beta_{P}},$$

(6) $$V_{S}=\alpha_{S}{\left(\frac{\sigma_{n}}{1kPa}\right)}^{\beta_{S}},$$

where *α* is the wave velocity at a 1 kPa confining stress, and the *β* exponent is the stress sensitivity. The subscripts P and S represent the longitudinal and shear wave velocities, respectively. Figure 4, Figure 5 and Figure 6 present the changes in longitudinal and shear wave velocities for different soil gouge fill conditions. The *α* factors and *β* exponents during loading and unloading are also listed. The longitudinal and shear wave velocities increased with increased axial stress for all tested specimens. Sand and clay gouge fill specimens displayed lower wave velocities compared with jointed rock specimens having clean joints for all tested gouge thicknesses and stress levels. This phenomenon is consistent with previous studies, where gouge-filled joints displayed lower stiffness compared with clean joints, making them more likely to be the weakest points in the jointed rock mass [25,26,27].

Thicker gouge fills resulted in more contribution of the gouge material to the overall stiffness of the equivalent continuum specimen. Both 2 and 5 mm thicknesses are more significant than the scale of rock asperities. However, the thicker gouge fill contributes more significantly to the overall stiffness of the equivalent continuum specimen. Sand gouge fill resulted in a decrease in longitudinal wave velocity of 32–42% for 2 mm gouge fill and 39–50% for 5 mm gouge fill specimens, with respect to the wave velocity of the clean-cut joint specimen during loading for each stress level. The decreases in shear wave velocity were 39–42% and 56–65% for 2 and 5 mm gouge fill specimens, respectively. Clay gouge fill resulted in similar decreases in both longitudinal and shear wave velocities. Decreases of 58–64% and 67–76% in longitudinal wave velocity were observed for 2 and 5 mm gouge fill specimens, respectively. The decreases in shear wave velocity were 60–62% and 61–71% for 2 and 5 mm gouge fill specimens, respectively. Smaller changes in wave velocity with gouge fill were observed at higher stress levels, implying that the increased confinement and resulting gouge fill compaction resulted in less impact of the gouge fill on the overall stiffness of the equivalent continuum specimen.

The longitudinal and shear wave velocities during unloading were higher for sand- and clay-filled jointed rock specimens, whereas no preloading effect was observed for the clean jointed rock specimen. The differences in wave velocity during loading and unloading for gouge-filled jointed rocks are similar to the typical soil response noted by Fratta and Santamarina (2002) [18]. Preloading causes permanent changes in the soil fabric and contact geometry, which affects wave propagation [28]. The soil gouge fill undergoes compaction and other mechanical changes in its matrix with an increase in axial stress and subsequent strain development. This is noted in the changes in the *α* factors and *β* exponents of Equations (5) and (6) during loading and unloading. The increase in *α* and decrease in *β* after loading for all tested specimens imply increased wave velocity and decreased stress sensitivity with gouge compaction. The effects of preloading were more apparent in soft clay gouges owing to their more compressive nature. The compressive nature of clay particles and contact restructuring during loading contributes to the increased stress sensitivity. This notable trend also implies a non-Hertzian contact for thick joint fills having soft cohesive gouge materials.

### 3.2. Effects of Gouge Thickness

**Figure 7:**
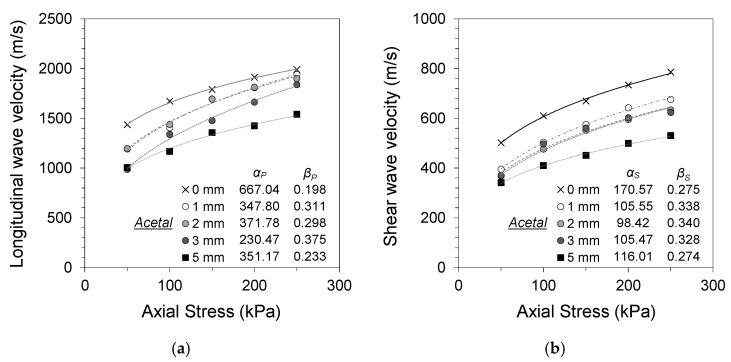
Specimen with acetal gouge (1, 2, 3, and 5 mm): (**a**) longitudinal wave velocity against normal stress; (**b**) shear wave velocity against normal stress.

Figure 7 presents the changes in longitudinal and shear wave velocities of jointed rocks with homogeneous isotropic acetal gouge fill at different fill thicknesses. Only the wave velocities during loading are shown, as acetal gouge fills displayed similar indifferences to preloading as with clean-cut joints. All acetal gouge specimens displayed lower wave velocities compared with the clean-cut joint specimens regardless of their fill thickness. The longitudinal and shear wave velocities decreased with increased gouge thickness for all tested axial stress levels. The average longitudinal wave decreased by 7.3%, 9.2%, 17.9%, and 26.5% for 1, 2, 3, and 5 mm gouge fill thicknesses, respectively, and the average shear wave velocity decreased by 15.9%, 19.4%, 20.9%, and 32.4% for 1, 2, 3, and 5 mm gouge fill thicknesses, respectively. Increased gouge thickness resulted in greater contribution of the less stiff acetal material to the overall stiffness of the equivalent specimen. This decrease in wave velocity was not as drastic as that of the soil gouge fill specimens owing to the comparatively smaller impedance difference between granite and acetal.

The impact of gouge fill thickness using acetal discs was greater on shear wave propagation compared with that on longitudinal waves. Longitudinal wave propagation in gouge-filled jointed rocks is affected by the effective contact area between the rock and gouge–rock interfaces and the gouge material properties [14,29]. With the acetal gouge specimens, only slight differences in the contact area occur with increased stress, as the clean-cut joint interfaces are identical regardless of the gouge fill thickness and do not experience changes in contact geometry. For shear wave propagation, the torsional friction between the rock and gouge interfaces and the stiffness of the gouge material contribute to the wave propagation characteristics. With increased gouge thickness and confinement, improved rock–gouge interface contacts result in a greater contribution of the acetal disc to the overall torsional stiffness of the equivalent continuum specimen. Hence, the wave propagation characteristics of the equivalent continuum specimen follow those of acetal.

## 4. Analysis

### 4.1. Hertzian Power Function Parameters

**Figure 8:**
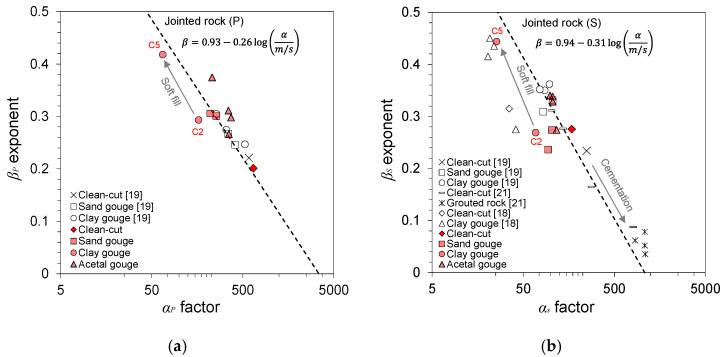
*α* and *β* parameters for the velocity–stress power relationship for different gouge fill materials and fill thicknesses. The trend line for jointed rock specimens was adopted from Cha et al. (2009) [19] and Cha et al. (2014) [30]. (**a**) *V_P_* parameters; (**b**) *V_S_* parameters.

The *α* factor and *β* exponent values are plotted with the fitted equation for jointed rocks from previous studies [19,30] in Figure 8. The *α* and *β* values of jointed, gouge-filled, and grouted rock specimens from previous studies are also plotted [18,19,21]. The longitudinal and shear wave velocities of all tested gouge specimens in this study showed a good fit with the fitted equation. The wave velocities displayed a general decrease in wave velocity with increased stress sensitivity to the gouge fill. This trend continued with increased gouge thickness and decreased gouge stiffness. The general distribution of points is indicative of this trend, where the homogeneous isotropic acetal fill displayed the largest wave velocity and smallest stress sensitivity, and the soil gouge fills displayed a gradual decrease in wave velocity and increase in stress sensitivity. Acetal and sand gouges showed minor differences in wave velocity and stress sensitivity with increased thickness, whereas clay gouge displayed a relatively large change between the 2 mm clay gouge (C2) and 5 mm clay gouge (C5). The low wave velocity and high stress sensitivity characteristics of clay gouge can be attributed to the non-Hertzian contact changes as the clay particles undergo compression and particle restructuring, leading to permanent changes in the soil fabric and contact geometry. Similar joint-dependent characteristics were observed for grouted rock joints [21]. Gouge filling and joint cementation with grouting lead to increased stress resistivity and improved elastic wave propagation. The velocity–stress power relationships reinforce the notion that the wave propagation characteristics in jointed rock masses are greatly dependent on the gouge conditions.

### 4.2. Absolute Gouge Thickness and Joint Stiffness

**Figure 9:**
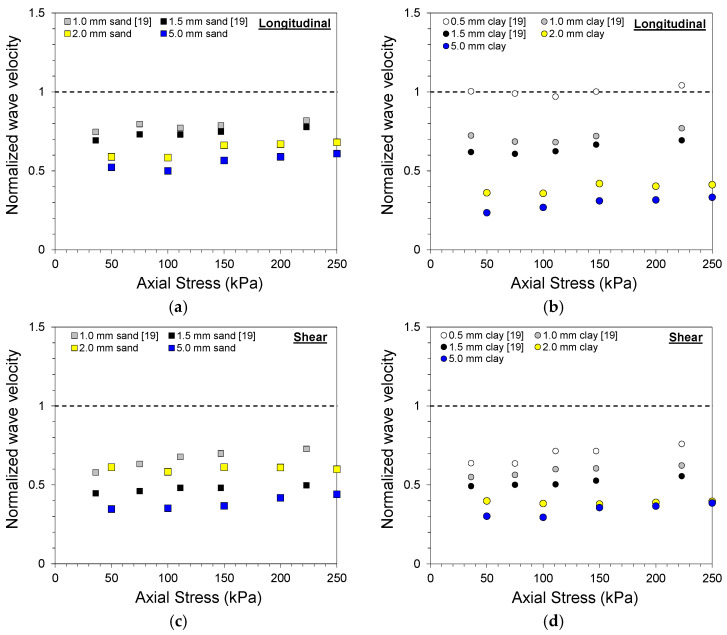
Normalized wave velocity for different gouge fill conditions: (**a**) longitudinal wave velocity of sand gouge fill; (**b**) longitudinal wave velocity of clay gouge fill; (**c**) shear wave velocity of sand gouge fill; (**d**) shear wave velocity of clay gouge fill.

The gouge fill thickness is often measured in the field for rock mass classification, such as the joint alteration number *J_a_* in the Q-system [31]. However, the use of the absolute gouge fill thickness as a quantitative indicator of joint properties is questionable and remains uncertain. The wave velocity of sand and clay gouge fill specimens was compared with previous QSRC test results on similar soil gouge fill specimens [19], as shown in Figure 9. The wave velocity of the clean-cut joint specimen for each axial stress level was used to normalize the wave velocity of sand and clay gouge fill specimens obtained during loading.

The wave velocities of the soil gouge specimens all displayed a decrease in normalized wave velocity with increased gouge fill thickness. A large initial drop in normalized wave velocity was observed with the presence of gouge fill, and a further decrease in normalized wave velocity was exhibited with increased gouge fill thickness. However, the amount of decrease in normalized wave velocity varied between the gouge fill material and type of wave. A large jump in normalized longitudinal wave velocity was observed between 0.5 and 1.0 mm, followed by another jump between 1.5 and 2.0 mm for clay gouge fill specimens in Figure 9b. The normalized shear wave velocities of sand gouge fill specimens in Figure 9c did not decrease in the order of absolute gouge fill thickness and displayed inconsistencies between the two datasets. This discrepancy can be attributed to the different surface conditions of the rock discs. In addition, the use of plastic membranes and the addition of a lever system for axial loading in this study could have affected the wave velocity measurements. Nevertheless, the aforementioned issues highlight the limitations of using the absolute gouge fill thickness as an indicator of jointed rock properties.

The spring constant of the joints in the form of joint stiffness can be considered an alternative method for characterizing joint characteristics [32]. The equivalent moduli of a jointed rock mass can be obtained from the longitudinal and shear wave velocities using the following equations:

(7) $$E_{eq}=\rho_{m}V_{P}^{2},$$

(8) $$G_{eq}=\rho_{m}V_{S}^{2},$$

where *p_m_* is the density of jointed rock mass. For a jointed rock system with regularly spaced joints, the normal joint stiffness (*k_n_*) and shear joint stiffness (*k_s_*) can be obtained from the equivalent moduli using the following equations:

(9) $$\frac{1}{E_{eq}}=\frac{1}{E}+\frac{1}{k_{n}S},$$

(10) $$\frac{1}{G_{eq}}=\frac{1}{G}+\frac{1}{k_{s}S},$$

where *S* is the joint spacing, *E* is the elastic modulus of intact rock, *G* is the shear modulus of intact rock, *E_eq_* is the equivalent elastic modulus of the jointed rock mass, and *G_eq_* is the equivalent shear modulus of the jointed rock mass. The normal and shear joint stiffnesses at 50 kPa axial stress were calculated using Equations (7)–(10) for each specimen and are summarized in Table 3. A clear decrease in normal and shear joint stiffnesses was observed with increased gouge thickness and decreased gouge material stiffness. The suggested joint stiffness values can be used as input parameters for numerical analysis on gouge-filled joints.

## 5. Conclusions

In this study, an experimental investigation was performed on the effects of gouge material and gouge thickness on the elastic wave propagation in jointed rock specimens. The main findings of this study are as follows:

1. Gouge fill resulted in decreased longitudinal and shear wave velocities compared with clean-cut joints owing to the decreased joint stiffness. The decrease in wave velocity depended on the applied axial stress and stiffness of the gouge fill material.
2. The increased axial stress resulted in higher wave velocities for all tested jointed rock specimens. The change in stress-dependent wave velocities indicates that the wave propagation characteristics are governed by the properties of the gouge material. The stress-dependent changes were more noticeable for thicker soil gouge fills.
3. The soil gouge specimens were susceptible to loading and unloading conditions. The initial preloading effects caused permanent changes in the soil fabric and contact geometry, similar to the typical soil response. The clay gouge also displayed a considerable discrepancy between the loading and unloading wave velocities compared with sand gouge owing to their more compressive nature. The effects of loading and unloading were negligible for the unfilled and homogeneous acetal gouge fills.
4. Longitudinal wave propagation is affected by the contact area between the rock and gouge-rock interfaces and the gouge material properties. Shear wave propagation is affected by the torsional friction between the rock-gouge interfaces and the stiffness of the gouge material.
5. The ranges of *α* (wave velocity at 1 kPa axial stress) and *β* (stress sensitivity) values of joint-filled rocks follow the general trend line outlined in previous studies. A general decrease in the 1 kPa wave velocity and increased stress sensitivity were observed with gouge filling. This trend continued with increased gouge thickness and decreased gouge stiffness. The low wave velocity and high stress sensitivity characteristics of the clay gouge indicate non-Hertzian contact changes.
6. The normal and shear joint stiffnesses calculated using the equivalent continuum model provide a quantitative indicator of the effects of gouge-filled joints and can be adopted as input parameters for numerical analysis.

Further studies on different gouge materials and consideration of strain-dependent wave propagation characteristics are needed to provide a more comprehensive analysis of the effects of gouge fill on jointed rock masses.

## Figures and Tables

**Table 1:** Properties of materials used in this study.

Rock Discs: Gneiss	
Dimensions	Disc thickness = 25 mm
	Inner diameter = 25 mm
	Outer diameter = 63 mm
Density	*ρ* = 2704 kg/m^3^
Intact Wave Velocity	*V_P_* = 4750 m/s
	*V_S_* = 3100 m/s
**Gouge Material: Jumunjin** **S** **and**	
Mean Particle Diameter	*D*_50_ = 421 μm
Specific Gravity	*G_S_* = 2.65
**Gouge Material: Kaolinite** **C** **lay**	
Mean Particle Diameter	*D*_50_ = 3 μm
Specific Gravity	*G_S_* = 2.70
**Gouge Material:** **A** **cetal Plastic**	
Dimensions	Disc thickness = 1, 2, 3, 5 mm
	Inner diameter = 25 mm
	Outer diameter = 63 mm
Density	*ρ* = 1451 kg/m^3^
Intact Wave Velocity ^1^	*V_P_* = 2480 m/s
	*V_S_* = 1090 m/s

^1^ Reference values from Asay and Guenther (1967) [24] on Delrin acetal nylon with similar densities at room temperature (25 °C).

**Table 2:** Summary of experimental test cases.

Configuration	Specimen Description	Specimen Image	Gouge Thickness
Effects of soil gouge fill	Clean-cut joints	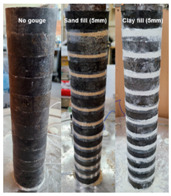	-
	Jumunjin sand gouge		2, 5 mm
	Kaolinite clay gouge		2, 5 mm
Effects of gouge fill thickness	Acetal plastic gouge	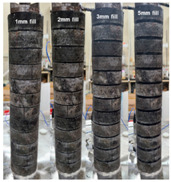	1, 2, 3, 5 mm

**Table 3:** Summary of normal and shear joint stiffness input values obtained from QSRC tests.

Gouge Material	Gouge Thickness (mm)	Normal Joint Stiffness (*k_n_*) (GPa/m)	Shear Joint Stiffness (*k_s_*) (GPa/m)
No Fill	0	235.32	26.94
Acetal	1	149.85	15.65
	2	141.84	12.84
	3	90.02	12.30
	5	86.73	9.36
Sand	2	69.91	8.88
	5	46.41	4.43
Clay	2	25.87	3.74
	5	9.27	1.80

## Data Availability

The data presented in this study are available on request from the corresponding author.
